# Comparative Evaluation of *Azadirachta indica* (Neem) Chip and Soft Tissue Diode Lasers as a Supplement to Phase I Periodontal Therapy in Localized Chronic Moderate Periodontitis: A Randomized Controlled Clinical Trial

**DOI:** 10.1155/2022/6109040

**Published:** 2022-06-15

**Authors:** Minal N. Ganvir, Simran R. Parwani, Dhanashree S. Chaudhary, Rajkumar Parwani, Himanshu Dadlani, Ashok K. Vikey, Kshipra P. Kawadkar, Nishita S. Jaju, Niccoló Giuseppe Armogida, Gianrico Spagnuolo

**Affiliations:** ^1^Department of Periodontology, V.Y.W.S. Dental College and Hospital, Amravati, Maharashtra, India; ^2^Department of Oral Pathology and Microbiology, V.Y.W.S. Dental College and Hospital, Amravati, Maharashtra, India; ^3^Kalka Dental College, Meerut 250016, India; ^4^Department of Oral Pathology and Microbiology, Government College of Dentistry, Sardar Patel Marg, Indore-452 001, Madhya Pradesh, India; ^5^Department of Neurosciences, Reproductive and Odontostomatological Sciences, University of Naples “Federico II”, Naples 80131, Italy; ^6^Institute of Dentistry, I. M. Sechenov First Moscow State Medical University, Moscow 119435, Russia

## Abstract

**Introduction:**

The current trial aimed to assess and compare the efficacy of neem chip and diode laser as a local drug delivery (LDD) agent as a supplement to phase I periodontal therapy in treatment of localized chronic moderate periodontitis. *Materials and Methodology*. Fourteen systemically healthy participants with 4–6 mm deep periodontal pockets at least in three quadrants (with no alveolar bony defect amenable to respective or regenerative osseous surgery, as seen in orthopantomograph) were selected for the trial. One week after phase I therapy, 10% absorbable chip of neem (commercially prepared by staff of a pharmacy college, Sheriguda, India) was placed in the periodontal pocket on one site, and soft tissue diode laser pocket sterilization was performed on the other site of the arch. Remaining one site was considered as a control. Parameters recorded clinically were plaque index (PI), papillary bleeding index (PBI), probing pocket depth (PPD), and relative attachment level (RAL) measured at baseline, 21^st^ day, and one month postoperatively.

**Results:**

Statistically significant improvements were observed in all clinical parameters at one month as compared to baseline for both treatment groups.

**Conclusion:**

Neem chip supplemented with phase I therapy showed best improvement in clinical parameters followed by laser supplemented with phase I therapy in comparison to phase I therapy alone at one month follow-up. *Clinical Significance*. Neem chips are nature's products, affordable without side effects, with a potential to be used as a local drug delivery agent in treating moderate chronic periodontitis.

## 1. Introduction

Chronic periodontitis is a condition characterized by inflammation of the supporting tissues of the teeth caused mainly by mixed microbial infection [1]. The deleterious effect of this type of periodontitis is not only restricted to the oral cavity but also possesses a risk for uncontrolled diabetes and heart disease [2]. Hence, early diagnosis of periodontal infection is necessary [3]. The purpose of periodontal treatment is to prevent, slow down (control), or eliminate periodontal disease and restore lost function, esthetics, and comfort [4]. The main goal of periodontal treatment is to restore the health of inflamed tissues, eliminate diseased pockets, and reduce the number of pathogenic microorganisms [5]. Surgical and nonsurgical treatments to remove plaque biofilm are important in the management of periodontal disease [6]. Although mechanical treatment with scaling and root planning (SRP) reduces the level of subgingival microbiota, it does not eradicate all the pathogens that reside deep in the connective tissue, mainly causing tissue destruction [7]. Systemic antibiotics are used to reduce the number of subgingival microflora. However, systemic therapy is commonly associated with side effects. In order to overcome the limitations of systemic antimicrobial therapy, a different approach has been developed using local delivery systems containing antibiotics or antiseptic agents [8]. The most effective local delivery agents include tetracycline fibers [9], 10% doxycycline [10, 11], 2% minocycline [12], metronidazole [13], and chlorhexidine gluconate [14], but none of them are devoid of side effects. To combat the side effects of these drugs, research is being done on the use of natural products. There have been various formulae under research that have increased our curiosity about the medicinal value of natural products such as turmeric, aloe, neem, tulsi, cork bark, and pomegranate; and all of these are widely tested these days [15]. Neem has been used as a popular tool to keep gums and teeth healthy. Various compounds such as nimbin, nimbidin, nimbidol, sodium nimbinate, and azadirachtin are also found in neem which act as anti-inflammatory, antipyretic, antihistaminic, antifungal, antimalarial, vasodilator, analgesic, antibacterial, and antiulcer agents [16, 17]. Laser treatment is also one of the most effective tools for reducing the burden of germs and inflammation by laser-assisted pocket sterilization, when used as a supplement to conventional therapies such as SRP. The primary cause of periodontal disease is bacterial infection; eliminating periodontal pathogenic microorganisms will fetch positive results. Toxins released from these bacteria are destroyed by laser power. SRP supplemented with laser treatment has been shown to improve oral health by reducing pocket depth [17]. Venilla et al. [18] showed successful benefits in clinical and microbiological parameters using the neem chip when delivered by local delivery of this agent in the periodontal pockets as compared to SRP alone. This clinical trial was designed to evaluate the effect of neem chip and diode laser therapy when delivered in periodontal pockets as an SRP supplement in the therapeutic regimen of localized moderate chronic periodontitis.

## 2. Materials and Methodology

### 2.1. Selection Criteria for Study Sample

This trial was performed at Periodontology Department of VYWS Dental College and Hospital, Amravati, Maharashtra, with Institutional Ethical Committee no. IEC/56 (D)/2020. Fourteen participants were with localized moderate chronic periodontitis (categorized according to 1999 AAP classification of periodontal diseases) [19] in 30–60 years systemically healthy patients, with presence of ≥15 teeth in the mouth. At least three (two test and one control) loci from three different quadrants of the mouth were randomly selected in every participant by the coin flip method. Teeth included had probing pocket depths (PPD) of 4–6 mm. Tobacco chewers, smokers, and those with a history of previous dental surgery or previous treatment with antibiotics in the last six months and patients with metallic pacemaker and known hypersensitivity to neem were excluded. All the patients were well informed about the treatment procedure, for which their written consent was obtained.

### 2.2. Study Design (Figure 1)

The research was a split mouth clinical trial with one month active timeline. PI, PBI, PPD, and RAL were obtained and noted at baseline, 21 days, and one month postoperatively. Probing pocket depth from marginal gingiva to the deepest point of pocket was recorded using the pressure-sensitive periodontal probe. Customized acrylic stents to serve as reference points for relative attachment levels (RALs) were prepared. Readings/measurements were done at baseline, three weeks, and one month postoperatively. Study samples were divided into three groups with the random sampling method. Group I (control group): treatment with SRP only. Group II: treatment with neem chip along with SRP. Group III: treatment with laser and SRP. Groups II and III were experimental groups.

### 2.3. Neem Chip Preparation

The neem chips were prepared in the Laboratory of Hydroxy Propyl Cellulose (HPC) Implants at Sree Dattha Institute of Pharmacy, Sheriguda. Ingredients are given in Table 1. Solvent mixture was transferred into a 100 ml beaker, magnetic bead was added, and mixture was kept on a magnetic stirrer. RPM was adjusted to 700. Required quantities of polyvinylpyrrolidone (PVP), sodium carboxy methyl cellulose, and polyethylene glycol (PEG) were transferred to the beaker and stirred until a uniform dispersion was obtained. To this mixture, required quantity of HPC was added little by little and stirring was continued until HPC was completely added. Polymer was stirred for 30 minutes until uniform polymer dispersion was obtained. Required quantity of neem extract was added to the polymer dispersion and stirred for 30 minutes. Whole polymer-neem dispersion was transferred to a clean Anumbra Petri dish. An inverted funnel was kept on the surface of the Petri dish and kept overnight for the evaporation of solvent. After one day, the funnel was removed and the polymer strip was peeled and wrapped into aluminium foil until further use.

Clinical parameters which included measurements obtained at baseline, 21 days, and one month posttreatment were as follows:

- Plaque index (Turesky et al., modification of Quigley–Hein index) [20]
- Papillary bleeding index (Muhlemann HR) [21]
- Probing pocket depth (PPD)
- Reference point on the acrylic stent to obtain relative distance from base of pocket to that point (RAL)

## 3. Results

### 3.1. Statistical Analysis

Intragroup comparison of change in PI, PBI, PPD, and RAL within each group was accomplished using the repeated measure ANOVA test. Pairwise comparison of change in PI, PBI, PPD, and RAL within each group was performed using the post hoc Bonferroni test. Intergroup comparison of all the variables between three groups was performed using the one-way ANOVA test. Intergroup comparison of all the variables between three groups was done using the post hoc Tukey test.

The measurements (PI, PBI, PPD, and RAL) were made by well-trained postgraduate students unaware of therapeutic procedures done in the participants.

**Figure 2:**
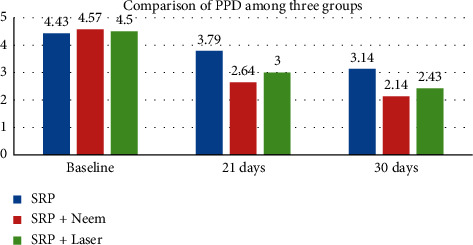
Graph of comparison of PPD among three groups.

When intragroup comparison was done, the PI score at baseline was 2.30 ± 0.37, 2.18 ± 0.35, and 2.18 ± 0.42 mm in SRP (control), neem (test group), and laser (test group), respectively. At 21 days, PI value reduced to 1.17 ± 0.47, 0.75 ± 0.20, and 0.94 ± 0.32, which further reduced at 30 days to 0.87 ± 0.33, 0.50 ± 0.14, and 0.63 ± 0.23 at one month. This was statistically significant in all groups from baseline to 30 days. The PBI score at baseline was 2.33 ± 0.35, 2.38 ± 0.27, and 2.26 ± 0.32 mm in SRP (control), neem (test group), and laser (test group), respectively. At 21 days, PBI score reduced to 0.97 ± 0.20, 0.63 ± 0.09, and 0.75 ± 0.11, which further reduced to 0.65 ± 0.17, 0.47 ± 0.10, and 0.52 ± 0.10 at one month. This was statistically significant in all groups from baseline to 30 days. The PPD score at baseline was l4.43 ± 0.51, 4.57 ± 0.51, and 4.50 ± 0.52 mm in SRP, neem, and laser groups, respectively. At 21 days, PPD score reduced to 3.79 ± 0.58, 2.64 ± 0.50, and 3.00 ± 0.56, which further reduced to 3.14 ± 0.54, 2.14 ± 0.36, and 0.43 ± 0.51 at one month. Overall comparison of PPD at baseline showed a statistically nonsignificant difference among three groups. PPD scores were least in the SRP plus neem group followed by the SRP plus laser group and then highest in the control group at one month. The difference between control and both test groups was statistically significant and that between both test groups was statistically insignificant (Table 2 and Figure 2).

**Figure 3:**
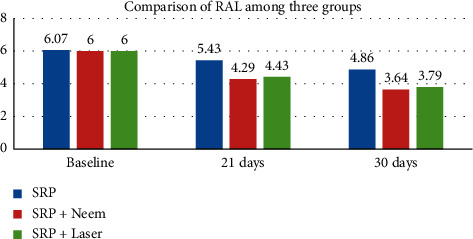
Graph of comparison of RAL among three groups.

The RAL score at baseline was 6.07 ± 0.73, 6.00 ± 0.56, and 6.00 ± 0.39 mm in SRP, neem, and laser groups, respectively. At 21 days, RAL score reduced to 5.43 ± 0.76, 4.29 ± 0.61, and 4.43 ± 0.52 mm which further reduced to 4.86 ± 0.54, 3.64 ± 0.50, and 3.79 ± 0.70 mm at one month (Table 3 and Figure 3).

Overall intergroup comparison of PI, PBI, PPD, and RAL scores at baseline showed a nonsignificant difference among all three groups. All scores were least in the SRP plus neem group followed by the SRP plus laser group and then highest in the control group at one month. The difference between control and both test groups was statistically significant and that between both test groups was statistically insignificant.

## 4. Discussion

Plaque is a major cause of gingivitis which is followed by periodontitis; so, till date, the basic treatment of periodontitis aimed at eliminating supra and subgingival plaque biofilm by mechanical debridement. In addition to this, regenerative methods like the use of autogenous platelet concentrates help to reduce moderate periodontal pocket depths while providing an inclusive treatment plan [22–24]. Oral rinses and irrigation systems are easily employed and commonly used, but the irrigation system is less effective in controlling moderate periodontitis, as the desired concentration of the drug is often not obtained in the periodontal pockets due to inadequate penetration of the drug in moderate periodontal pockets. One of the FDA-approved available LDD agents in the market which is considered a gold standard in the treatment of moderate periodontitis contains 2.5 mg chlorhexidine gluconate (PerioChip) [25, 26], but is expensive, synthetic, and not easily available. Keeping this perspective in mind, a randomized clinical trial was designed to compare the effects of the neem chip (economical, herbal, and readily available) agent as a local drug delivery agent and pocket sterilization with soft tissue diode lasers as compared to SRP alone in moderate depth periodontal pockets of chronic periodontitis. Clinical parameters (PI, PBI, PPD, and RAL) were assessed at baseline, 21 days, and 30 days. In the current study, all four parameters showed significant improvement from baseline to 30 days in all three groups. When compared between the groups, there was a significant difference between the control and test groups and a significant difference between the two test groups for all parameters. The plaque index (PI) scores were reduced from baseline and remained very low (<1) until the end of the one month study period following the monitoring of test and control groups. This was the result of reiterated oral hygiene directions given to participants throughout the study. Least scores were observed for the SRP + neem group followed by the SRP + laser group and highest scores were with only the SRP group. This may be attributed to the antimicrobial property of neem.

Results of the current trial are in line with the study carried by Vennila et al. [18] where they found significantly reduced plaque accumulation on days 7 and 21 and 3 months with the use of neem chip.

Jain and colleagues [27] in a clinical trial compared gingivitis index with neem chip plus SRP and SRP alone, and their results were also similar with the current study at four weeks.

Similarly, significant pocket depth reduction in both test and control groups of our study was comparable with the findings from the study carried by Saini et al. [28] where they evaluated the efficacy of herbal LDD agents as a supplement to SRP and SRP alone in the treatment plan of chronic periodontitis. They compared three groups: SRP plus neem chip, SRP plus turmeric chip, and SRP alone for a period of 3 months. Their residual pocket depths after three months were 3.919 ± 0.85, 4.18 ± 0.69, and 4.32 ± 0.65 for neem, turmeric, and control groups, respectively.

Furthermore, significantly greater reduction of PPD obtained in the test group vs. the control group of our study is in accordance with the study carried by Mehta et al. [29] where they found significantly greater reduction in probing pocket depths with the SRP + neem group followed by the SRP + turmeric group compared to the SRP group only.

In terms of use of soft tissue diode lasers, our trial exhibited similar results as found by studies conducted by Dukic et al. [30] and Crispino et al. [31] that showed a significant improvement in GI, PI, PPD, and RAL when SRP + laser was compared to SRP alone.

On the other hand, results of the present study in terms of soft tissue diode lasers were contrary to those achieved by Jose et al. [32] and De Micheli et al. [33] where nonsignificant improvement in pocket depths and clinical attachment gain in the laser group were availed when SRP + laser therapy was compared to SRP alone.

In the current study, both test agents SRP + neem and SRP + lasers were shown to be efficient in improving clinical parameters such as PI, GI, PPD, and RAL; although, the SRP + neem group exhibited best results, followed by SRP + lasers and then SRP alone.

## 5. Conclusion

Neem and soft tissue diode laser therapy as LDD being adjuncts to SRP are more efficient in improving chronic periodontitis parameters such as plaque index, gingivitis index, probing pocket depths, and relative clinical attachment levels vs. SRP alone in a short term of one month. Best results were observed with the insertion of the neem chip in periodontal pockets, which may be attributable to the antimicrobial nature of neem. The long-term effects of these agents, however, are still arguable. Furthermore, studies with larger sample size and longer follow-up periods may aid in providing a greater insight to the results of these therapies.

## Figures and Tables

**Figure 1:**
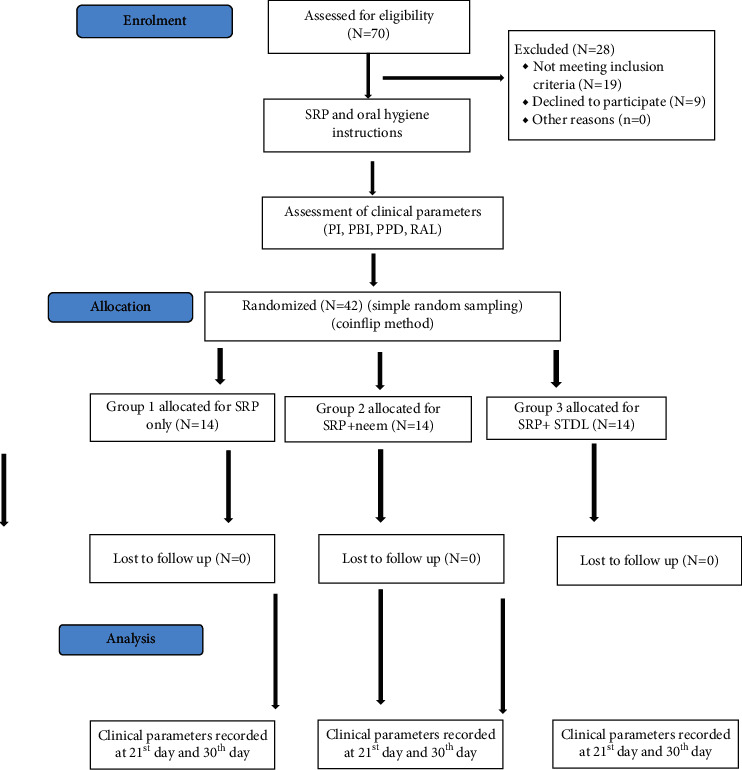
Consort flow diagram.

**Table 1:** Neem chip formulation.

Ingredients	Quantity

Neem	10%
Hydroxy propyl cellulose	800 mg
Sodium carboxy methyl cellulose	200 mg
Polyvinylpyrrolidone	100 mg
Polyethylene glycol	100 mg
Ethanol/water	10 ml

**Table 2:** Intragroup comparison of change in PPD within each group.

Variable	Baseline	21 days	30 days	*P* value

SRP	4.43 ± 0.51	3.79 ± 0.58	3.14 ± 0.54	0.001^*∗*^
SRP + neem	4.57 ± 0.51	2.64 ± 0.50	2.14 ± 0.36	0.001^*∗*^
SRP + laser	4.50 ± 0.52	3.00 ± 0.56	2.43 ± 0.51	0.001^*∗*^

Repeated measure ANOVA test. ^*∗*^Significant difference at *p* ≤ 0.05.

**Table 3:** Intragroup comparison of change in RAL within each group.

Variable	Baseline	21 days	30 days	*P* value

SRP	6.07 ± 0.73	5.43 ± 0.76	4.86 ± 0.54	0.001^*∗*^
SRP + neem	6.00 ± 0.56	4.29 ± 0.61	3.64 ± 0.50	0.001^*∗*^
SRP + laser	6.00 ± 0.39	4.43 ± 0.52	3.79 ± 0.70	0.001^*∗*^

Repeated measure ANOVA test. ^*∗*^Significant difference at *p* ≤ 0.05.

## Data Availability

The data used to support the findings of this study are available from the corresponding author upon request.

## Additional Points

Clinical parameters showed statistically significant improvement in neem the chip + SRP group followed by the laser + SRP group as compared to SRP alone.
